# Supplementary material for: Antibiotic-Induced Gut Microbial Dysbiosis Reduces the Growth of Weaning Rats via FXR-Mediated Hepatic IGF-2 Inhibition
Source: Nutrients. 2024 May 27;16(11):1644. doi: 10.3390/nu16111644 (PMC11175069; doi:10.3390/nu16111644)
Supplement: Supplementary file 1 [file nutrients-16-01644-s001.zip › nutrients-2988151-supplementary.pdf]

Table S1. Nucleotide sequences of specific primers

Target genes	GenBank accession	Primer sequences (5' to 3')	PCR product (bp)
IGF-1	NM_001082477.2	F: ACGCTCTTCAGTTCGTGTGT	161
		R: CTTCAGCGGAGCACAGTACA	
IGF-2	NM_001190162.1	F: GTACTTCCGGACGACTTCCC	187
		R: GTAACACGATCAGGGGACGG	
IGF-1R	NM_001414181.1	F: GCCCATGTGTGAGAAGACCA	216
		R: ACGCACACGCCTTTGTAGTA	
Gcg	NM_012707.3	F: GACAAACGCCATTCACAGGG	112
		R: TGTTCCGGTTCCTCTTGGTG	
DPP-4	NM_012789.2	F: CCCAGTTTAACGACACCGGA	237
		R: AGGCCACGTCACACAAGTAG	
GLP-1R	NM_012728.2	F: TTCATCAAAGACGCTGCCCT	262
		R: TAACGAACAGCAGCGGAACT	
AgRP	NM_033650.1	F: AGCAGACCGAGCAGAAGATG	129
		R: TGTTGTCCCAAGCAGGACTC	
NPY	NM_012614.2	F: CGCTCTGCGACACTACATCA	113
		R: TGGGGGCATTTTCTGTGCTT	
POMC	NM_139326.3	F: CCATAGACGTGTGGAGCTGG	147
		R: AGGGCTGTTCATCTCCGTTG	
CART	NM_017110.1	F: GGACATCTACTCTGCCGTGG	95
		R: GCGCTTCAATCTGCAACACA	
PPIA	NM_017101.1	F: AGGATTCATGTGCCAGGGTG	187
		R: CTCAGTCTTGGCAGTGCAGA	
IGF-2 Fragment1		F: TCTATCAGGCACCAAAGCCC	
		R: TCTCTACCACCAAGCAAGGC	
IGF-2 Fragment2		F: TCGGAAGAGAATCTTGGGCAC	
		R: ACCTTCCCCTTTTCAGAGACT	
IGF-2 Fragment3		F: AAACTGCATGGTCCCGCTAA	
		R: GGCATGCACCAAGGGAAAAA	
